# Dissecting the genetic structure and admixture of four geographical Malay populations

**DOI:** 10.1038/srep14375

**Published:** 2015-09-23

**Authors:** Lian Deng, Boon-Peng Hoh, Dongsheng Lu, Woei-Yuh Saw, Rick Twee-Hee Ong, Anuradhani Kasturiratne, H. Janaka de Silva, Bin Alwi Zilfalil, Norihiro Kato, Ananda R. Wickremasinghe, Yik-Ying Teo, Shuhua Xu

**Affiliations:** 1 Chinese Academy of Sciences (CAS) Key Laboratory of Computational Biology, Max Planck Independent Research Group on Population Genomics, CAS-MPG Partner Institute for Computational Biology (PICB), Shanghai Institutes for Biological Sciences, Chinese Academy of Sciences, Shanghai 200031, China; 2 Faculty of Medicine and Health Sciences, UCSI University, Jalan Merana Gading, Taman Connought, 56000, Kuala Lumpur, Malaysia; 3 Saw Swee Hock School of Public Health, National University of Singapore, Singapore; 4 Life Sciences Institute, National University of Singapore, Singapore; 5 Department of Public Health, Faculty of Medicine, University of Kelaniya, Ragama 11010, Sri Lanka; 6 Department of Medicine, Faculty of Medicine, University of Kelaniya, Ragama 11010, Sri Lanka; 7 Department of Pediatrics, School of Medical Sciences, Universiti Sains Malaysia, Kelantan 16150, Malaysia; 8 Department of Gene Diagnostics and Therapeutics, National Center for Global Health and Medicine, Tokyo 1628655, Japan; 9 NUS Graduate School for Integrative Science and Engineering, National University of Singapore, Singapore; 10 Genome Institute of Singapore, Agency for Science, Technology and Research, Singapore; 11 Department of Statistics and Applied Probability, National University of Singapore, Singapore; 12 School of Life Science and Technology, ShanghaiTec University, Shanghai 200031, China; 13 Collaborative Innovation Center of Genetics and Development, Shanghai 200438, China

## Abstract

The Malay people are an important ethnic composition in Southeast Asia, but their genetic make-up and population structure remain poorly studied. Here we conducted a genome-wide study of four geographical Malay populations: Peninsular Malaysian Malay (PMM), Singaporean Malay (SGM), Indonesian Malay (IDM) and Sri Lankan Malay (SLM). All the four Malay populations showed substantial admixture with multiple ancestries. We identified four major ancestral components in Malay populations: Austronesian (17%–62%), Proto-Malay (15%–31%), East Asian (4%–16%) and South Asian (3%–34%). Approximately 34% of the genetic makeup of SLM is of South Asian ancestry, resulting in its distinct genetic pattern compared with the other three Malay populations. Besides, substantial differentiation was observed between the Malay populations from the north and the south, and between those from the west and the east. In summary, this study revealed that the genetic identity of the Malays comprises a mixed entity of multiple ancestries represented by Austronesian, Proto-Malay, East Asian and South Asian, with most of the admixture events estimated to have occurred 175 to 1,500 years ago, which in turn suggests that geographical isolation and independent admixture have significantly shaped the genetic architectures and the diversity of the Malay populations.

The Malay people, who generally inhabit the Malay Archipelago, particularly the Peninsular Malaysia, is a group of genetically diverse populations^1^. As a major ethnic group settling in such a strategic hub in Southeast Asia, the Malay population may provide evidence for the complex historical background and demographic information in this region, which has never been fully understood to date.

In Malaysia, the Malays consist of several sub-groups, e.g., Melayu Minang, Melayu Jawa, Melayu Bugis and Melayu Kelantan, based on their respective geographical origins. Malay sub-groups have various historical origins and genetic affinities^1^,^2^. Both ancient and recent human activities have introduced gene flows from other populations into the Malays^3^,^4^,^5^. The indigenous groups (Orang Asli) in Malaysia, including Proto-Malay, Senoi and Negrito, interacted with the Malays because their habitats were situated adjacent to one another. The Malays are genetically related to the Orang Asli despite differences in their physical features^5^. For example, Negritos have short stature and very dark skin, which are more similar to the phenotypes of African Pygmies than to those of other Southeast Asian populations^6^,^7^. In addition, populations from other regions of Asia, even from other continents, have cultural and genetic influences on the Malays to various extents. In particular, Chinese, Indians, Arabians, and Europeans have substantially influenced the region since ancient times, and have had a more considerable impact in recent centuries due to the colonization and globalization of the entire human society, thus leading to the mosaic genomic pattern in the Malays^2^,^5^,^8^. Besides Malaysia, the Malays are distributed in the surrounding islands, e.g. Peninsular Indo-China, Singapore, Java, and Sri Lanka^9^. Malay populations share a common origin with other Austronesian populations^9^. During the 1400s and 1500s, the Malacca Sultanate was established and subsequently dominated the entire Malay Archipelago, and the Malay language was established as the official language in that region, replacing Sanskrit. The Malays, especially those in the western and southern Malaysia, have had frequent interactions with other Austronesian populations through trading or other social activities^10^. These early contacts did not result in a large-scale of population admixture, but intermarriage did exist. Gradually, the Malays mixed with the local residents in the surrounding islands, and have developed into various Malay ethnic groups^2^. These Malay ethnic groups affect not only the demography but also the culture, including religions and languages, outside Malaysia. However, the Malay people have been largely under-represented in studies on human genetic diversity and are not on the population list of large-scale international projects, such as the the International Haplotype Map Project^11^ (HapMap, http://www.hapmap.org), the Human Genome Diversity Project^12^ (HGDP, http://www.hagsc.org/hgdp) and the 1000 Genomes Project^13^ (http://www.1000genomes.org). Some recent studies on Asian populations have included a few Malay samples from Malaysia, Indonesia and Singapore^8^,^14^,^15^, but other Malay populations residing outside of Southeast Asia such as those living in Sri Lanka, have not been well studied.

The present study aimed to investigate the genetic relationship and population structure of the Malays from both Southeast Asia and South Asia to extend our knowledge about population history and the genetic landscape of human populations in Southeast Asia. Analyses were conducted by using 288,660 single nucleotide polymorphisms (SNPs) shared by 133 Malay individuals from Peninsular Malaysia, Singapore and Sri Lanka (abbreviated to PMM, SGM and SLM, respectively), as well as approximately 50,000 SNPs shared by 12 Indonesian Malays (IDM) located in Sumatra Island, 30 SGM and 38 PMM (20 Minangkabau, or locally known as Melayu Minang, a sub-tribe of Malay; 18 Kelantan Malay, or locally known as Melayu Kelantan) from the HUGO PanAsia SNP Consortium (PASNP)^8^. We also integrated 3,170 individuals from 90 worldwide populations with our data to obtain a higher resolution in deciphering the genetic diversity of the Malay populations. Genetic structure investigation was based on model-free methods that provided direct information on population relationships and the genetic makeup. Furthermore, we inferred the population admixture history of each Malay population to illustrate the population genetic structure patterns.

## Results

### Population genetic relationships

**Figure 1:**
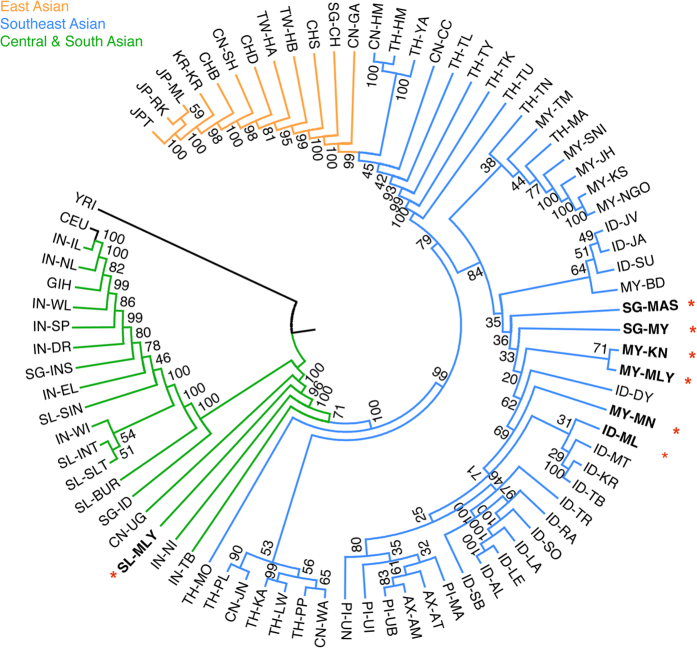
Phylogenetic tree showing genetic relatedness of four Malay populations. A phylogenetic tree was constructed by using the neighbor-joining method, taking YRI as the outgroup. The pair-wise population distance was measured by global *F*_ST_ with 1,000 bootstrapping repeats. Bootstrap values are noted on the branches. Population IDs are shown in Supplementary Table S4. Geographical groups are indicated by colors. The four Malay populations are highlighted in bold font with red asterisks.

As shown in the population phylogenetic tree (Fig. 1, Supplementary Figure S1), populations from closer geographical areas showed greater genetic similarities and were thus clustered. Among the four Malay groups, SGM, PMM and IDM were closely clustered and shared a distinct clade with other Southeast Asians, whereas IDM showed a closer relationship to populations from Sumatra and Java than to other Southeast Asians. On the other hand, SLM was lying on a cline of Southeast Asian and South Asian. Based on the genetic distance measured by *F*_ST_, SLM showed a closer relationship with South Asian populations (*F*_ST_ = 0.004–0.024) than with Southeast Asians (*F*_ST_ = 0.010–0.057). In addition, the genetic difference between SLM and the other Sri Lankan ethnic groups (*F*_ST_ = 0.007–0.012) was larger than that between any other Sri Lankan ethnic groups (*F*_ST_ = 0.001–0.003).

**Figure 2:**
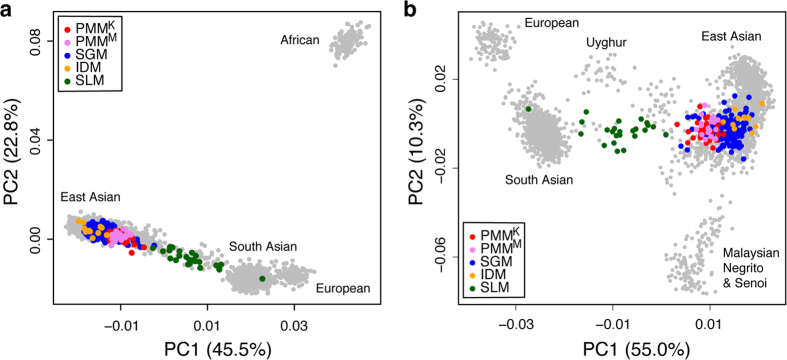
Plots of the first two principal components for hierarchical analyses of worldwide populations. (**A**) 2,713 individuals representing 83 populations from Africa, Europe and Asia. (**B**) 2,597 individuals representing 82 populations from Europe and Asia (excluding YRI). PMM^K^ and PMM^M^ denote Kelantan Malay and Minangkabau, respectively.

Similar population relationships were observed by using principal component analysis (PCA). At the global scale, the four Malay groups were considerably distinct from Africans, and similar to other non-Africans (Fig. 2a). South Asians (Indians and Sri Lankans) constituted a bridge between the Europeans and other Asian populations (Fig. 2). SLM, as well as the Uyghurs that have been proven to be an admixed population^16^, were situated between other Sri Lankan populations and Southeast Asians (Fig. 2). In general, the geographical distribution of these populations was fully reflected by their genetic relationships, although exceptions existed. However, PMM showed closer affinity to the Southeast Asian populations located outside Malaysia despite that they inhabited the adjacent regions of Peninsular Malaysia, possibly due to the population isolation and local adaptation of the Orang Asli populations, which in turn resulted from their special living environment and demographic history^5^ (Fig. 2b). By gradually excluding the populations less closely related to the Malays (Supplementary Figure S2), we finally identified that the western Indonesians had the closest relationship to PMM, SGM and IDM, followed by Filipino, Thai, Proto-Malay and Bidayuh (a native population from southern Sarawak). Furthermore, population differentiation was also observed between Malay populations from the north (SLM and Kelantan Malay) and the south (Minangkabau, SGM and IDM) (Pearson product-moment correlation coefficient (PCC) = 0.781 between the PCA coordinates and the geographical latitude, p-value < 2.2 × 10^−16^), and between those from the west (SLM) and the east (PMM, SGM and IDM) (PCC = 0.9 between the PCA coordinates and the geographical longitude, p-value < 2.2 × 10^−16^) (Supplementary Figure S3).

### Multiple population admixture in Malays

**Figure 3:**
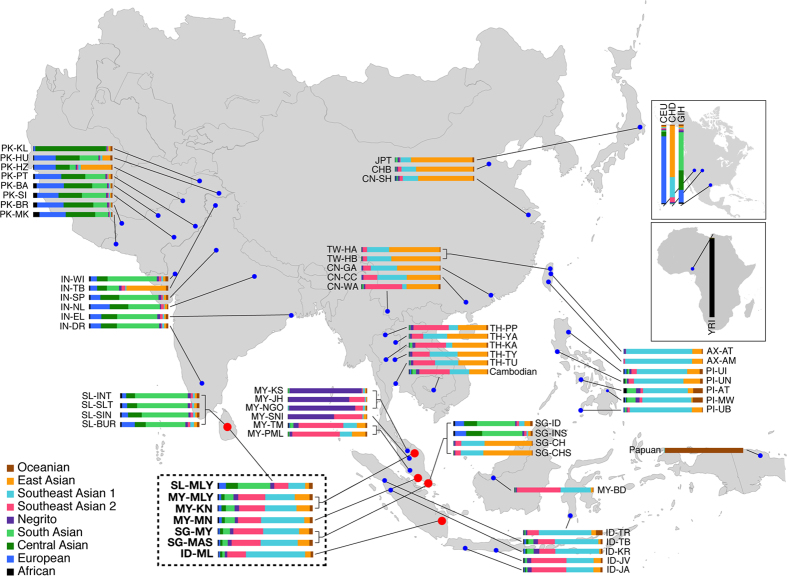
Locations and genetic makeup of the Malays and other populations. The averaged genetic makeup across individuals of each population are indicated by the bars. Each color represents an independent cluster at K = 9. Southeast Asian 1 and Southeast Asian 2 represent the aboriginal Southeast Asian component and Austronesian component, respectively. All the Malay populations are arranged in the dashed box. Population IDs are shown in Supplementary Table S4. The map is generated using the packages of R v2.11.1, including mapdata v2.2-3, mapplots v1.5 and maps 2.3-9 (http://cran.r-project.org/web/packages/).

An unsupervised ADMIXTURE analysis, without prior information of individual ancestry, was performed to investigate patterns of admixture in Asia. Nine ancestral clades were assumed to reflect the major genetic components in Asia (Fig. 3, Supplementary Figure S4). Collectively, South Asians and East Asians had their distinct genetic patterns. European, Central Asian and South Asian components predominated the South Asian genomes, whereas a large proportion of the East Asian component and a small proportion of the Southeast Asian component comprised the East Asian genomes. However, the Southeast Asian populations had more complex and diverse genetic makeup than the South Asians and the East Asians. Malay populations were admixed with multiple ancestries with no representative component identified in them, but most Malays had a genetic pattern distinct from other Southeast Asian populations.

Malay populations shared four major components, e.g., East Asian, South Asian, Austronesian and aboriginal Southeast Asian. East Asians, which contribute 4%–16% of the Malay genomes, had interactions with Malays at very recent time (100–200 years ago, assuming a single generation time of 25 years was applied throughout this study) (Table 1, Supplementary Table S1). We observed a substantial proportion of South Asian ancestry in the Malay populations as well, ranging from 3% to 34%, with the greatest proportion observed in the SLM and the most ancient admixture date in the PMM (625–1,400 years ago) and IDM (1,350–2,250 years ago). In addition, the proportions of Austronesian admixture (represented by the Taiwanese aborigines; labeled as Southeast Asian 1 in Fig. 3) and aboriginal Southeast Asian admixture (represented by Proto-Malays; labeled as Southeast Asian 2 in Fig. 3) were within the ranges of 15%–31% and 17%–62%, respectively, in the Malay populations, with a greater proportion in PMM, SGM and IDM. We observed nearly constant proportions (<5%) of components from African, European, hunter-gatherer (represented by Malaysian Negrito), and Oceanian (represented by Papuan) in most of the Southeast Asians examined in this study. However, these were disregarded in our interpretation because these could have resulted from the ascertainment bias. The time of admixture was estimated by using ALDER throughout the present study (see Materials and Methods).

While the genetic makeup of the four Malay populations shared similar ancestral components, the proportion of these components in each population substantially differed. In PMM, SGM, and IDM, the Austronesian and Southeast Asian aboriginal components had larger proportions than the East Asian and South Asian components. Specifically, very little genetic difference was found between PMM and SGM, in which the two major components were of nearly equal proportions and together comprised 60%–70% of the entire genome. IDM also showed a very similar genetic pattern, but was more marginally affected by the Austronesian population (Austronesian component: 62% in IDM, 31%–45% in SGM and PPM). However, the genetic admixture pattern of SLM was unique compared to the other Malay populations, exhibiting admixture between South Asians and Malays. The proportions of different components were relatively more comparable in SLM. For instance, the proportions of Central Asian and South Asian components in SLM were intermediate between those observed in Southeast Asians and South Asians, and a similar pattern was detected for the Southeast Asian component (Austronesians and Proto-Malays) and East Asian component.

To quantify the contribution of each ancestral component to the differentiation of Malay populations, we calculated the correlation between the proportions of each ancestral component and the values along PC1 of the 4 Malay populations in Supplementary Figure S2a. Supplementary Table S2 showed that all the four major components in the Malays were correlated with PC1 to different extents (p < 0.01). For example, the South Asian component was most highly correlated with the Malay differentiation (PCC = 0.927, p-value < 2.2 × 10^−16^), whereas the East Asian component showed a much less significant correlation (PCC = 0.268, p-value = 1.03 × 10^−4^). Furthermore, the European and Central Asian components, although they were not major components in the Malays, also largely contributed to the Malay differentiation (PCC = 0.807 and PCC = 0.579, respectively).

## Discussion

In this study, we conducted a genome-wide analysis of four geographical Malay populations from Malaysia, Singapore, Indonesia and Sri Lanka. We have analyzed the genetic diversity of the Malay populations and revealed substantial population differentiation and gene flow among these different geographical populations.

In general, all the four Malay populations were inferred to be substantially admixed with multiple ancestries from East Asian, South Asian, Austronesian, and Southeast Asian aboriginal people around 175–1,500 years ago (Fig. 3, Table 1), which could be attributable to their complex origins and frequent interactions with surrounding ethnic groups, as reported in historical records. As early as 2,000 years ago, several ancient Malay states emerged in the coastal areas of the Malay Peninsula, Sumatra, western Java and western Borneo. During the 7^th^–13^th^ centuries, the Malay culture reached its golden age, and influenced the entire Malay Archipelago^2^,^17^. In the 15^th^ century, the Malacca Sultanate initiated a major revolution in Malay history by promulgating its languages, religions and traditions, which has also recently influenced the immigrants from other countries^2^. Since the year 1299, the advent of the Kingdom of Singapura, a small Malay kingdom, in the modern-day island nation Singapore started the inhabitation of Malays in Singapore^18^. Although Sri Lanka and Southeast Asian countries have had very early interactions on politics, religion and culture since the 11^th^ century^19^, the earliest Malay inhabitants of Sri Lanka were brought in laborers from Indonesia and Malaysia by the Dutch and British colonizers during the 16^th^–17^th^ century^20^,^21^. The present-day SLM, which comprise 0.3% of the Sri Lankan population, are the descendants of the mixture of the local Sri Lankan people with those Malays^20^. Meanwhile, the first millennium of the Common Era also witnessed the trade migration of South Asians into the Malay kingdoms, as well as the arrival of Chinese, Arabian and Persian merchants in Island Southeast Asia^2^,^22^. From 1500s towards the end of the 20^th^ century, European countries, including Portugal, Netherlands, and Britain, colonized the majority of Southeast and South Asian countries, bringing about mating and gene flow across continents. More recently, due to the development of transportation and the globalization of the entire human society, the Southeast and South Asian populations have thus had more frequent gene flow from each other and from their surrounding neighborhoods, e.g., East Asians. Although previous genetic studies investigated less comprehensive Malay samples covering a smaller geographical range of Malay, the results indeed concurred with our findings of extensive genetic diversity among the Malays^1^,^8^.

The present study provides the first identification of the distinct genetic makeup of SLM compared to the other three Malay populations. SLM inherited much more South Asian ancestry, whereas PMM, SGM, and IDM are genetically similar to most of the other Southeast Asian populations, with minor fluctuations in the proportions of each genetic component across populations. Two factors have facilitated to shaping SLM to be a genetically intermediate population between South Asians and Southeast Asians (Figs 1 and 2). First, SLM originated from the admixture of Sri Lankan populations and the Malays. Assuming the Sri Lankan ethnic groups and Malay to be the ancestral populations, we identified 55%–61% of Sri Lankan component and 39%–45% of Malay component in SLM (Supplementary Table S3). Second, Sri Lanka is located in the strategic region bridging South Asia and Southeast Asia, and thus has been an important trading center as well as a melting pot of various ethnicities.

Unlike those isolated or ancient populations in Southeast Asia (e.g., Negritos), the Malays do not have a representative genetic component. Instead, four major ancestral components constitute the Malay genomes, making the Malays distinguishable from most of the other Southeast Asian populations (Fig. 3). The Austronesian and Southeast Asian aborigines have been determined to be the predominant components. As indicated by our results, aboriginal Taiwanese (Ami and Atayal) and Proto-Malays have the largest proportion of these two components; however, we were not able to determine the origin of the components because these could have been derived from any other source populations that have yet to be identified by our analyses. The genetic pattern of the Austronesian component across Southeast Asians follows the Austronesian migration route supported in the “Out of Taiwan” hypothesis^23^,^24^,^25^, which influenced the eastern part of Southeast Asian more than the western part of it. However, the Proto-Malay component, which has been reported to be an ancient Southeast Asian component^26^, is centered in the west. Considering that the Proto-Malays are an isolated aboriginal population in this region, it is likely that the Proto-Malay genetic component in other Southeast Asian populations, especially those located outside Peninsular Malaysia, was obtained from the broadly migrated Malay population, which had very close interactions with Proto-Malays. This observation also has important implications to the human migration history in Southeast Asia. Apart from the four major components, we observed small amount of other components in the Malays. These components were not included in our interpretation because they could be resulted from ascertainment bias. In ADMIXTURE analyses, we only used SNPs shared by global populations, leading to the reduction of the number of rare SNPs in the SNP panel of each population, thus providing less population-specific information. In this case, some genomic regions could be origin-ambiguous and have been assigned to multiple continental populations. The major human migration events that occurred in Southeast Asia may have geographical limitations, which means that different populations obtained the major genetic contributions from their respective close neighbors, thus leading to a correlation between geographical coordinates (both latitude and longitude) and population differentiation. This indicates the effect of geographical independency in human genetics. Even so, we did not rule out other migration events that would have had a smaller genetic impact. These findings may provide crucial clues to the peopling in Southeast Asia.

The major limitation of the present study lies in the possible bias of the estimated admixture time and fraction. For some populations, the sample size and SNP density were not highly appropriate for use in the analyses. On one hand, most of the Southeast Asian genotypes, including IDM, from PASNP^8^, only harbored 50,000 SNPs and comprised less than 20 individuals. On the other hand, we have integrated data from various international projects and genotyping platforms hence the number of SNPs in the combined datasets that were then applied to different analyses is very small. In this case, although PCA, genetic component analysis and the construction of population phylogenetic tree are sufficiently robust to fluctuations in sample size or SNP density, the estimation of admixture time and fraction, which were based on the calculation of linkage disequilibrium (LD) decay, might be highly accurate. Therefore, a more comprehensive collection of larger numbers of samples and SNP markers will be necessary in the future studies. Besides, the existing methods for admixture analysis are not powerful enough to handle the various Southeast Asian ethnic groups with complex historical and genetic backgrounds. This can be explained by two factors. First, our estimation was based on the hybrid isolation (HI) model^27^, which might not be representative of the real cases. Second, the populations referred to as the ancestries also recently obtained genetic materials from other populations, which could bias the estimation of the impact of ancestral populations on their admixed descendants. Therefore, more sophisticated methods may better facilitate the construction of human migration history in Southeast Asia.

In summary, the present study provides the first demonstration of the genetic relationship and genetic makeup of various Malay populations and indicated a complex human population admixture history in Southeast Asia. We propose that geographical isolation and independent admixture history have significantly shaped the genetic architectures and the diversity of the Malay populations. An important direction for future work is to extend to a wider coverage of Malay groups and increase the SNP density, to allow a more detailed investigation of the population genetic diversity in Southeast Asia.

## Methods

### Populations and samples

The present study focused on 213 unrelated Malay individuals from 4 Malay groups that were collected from different datasets. All the 27 SLM samples, as well as 538 Sri Lankan samples from other ethnic groups (303 Tamil, of which 200 were of Indian ancestry, 35 Burger and 200 Sinhalese) were genotyped on an Illumina 2.5 M array. The other 186 Malay samples were previously reported, including 89 SGMs from the Singapore Genome Variation Project^14^ (SGVP, http://www.statgen.nus.edu.sg/~SGVP/), 30 SGMs, 12 IDMs and 38 PMMs from PASNP^8^, and 17 PMMs as reported in Deng *et al.* (2014)^5^. Notably, the PMM population in the present study consisted of two subgroups: 35 Kelantan Malays and 20 Minangkabau.

To characterize the genetic variation of the 4 Malay groups on a global scale, we also included 3,170 unrelated individuals from 90 worldwide populations reported in HapMap Phase 3^11^, HGDP^12^, SGVP^14^, PASNP^8^ and our previous study^5^. The details of sampled populations are summarized in Supplementary Table S4.

### Sample and SNP quality control

Our analyses excluded samples with missingness greater than 5%. Then, we used PLINK v1.07^28^ (http://pngu.mgh.harvard.edu/~purcell/plink/) to control the data quality. Stringent sample filtering was conducted within each of the examined populations, and in total, 113 samples with missing rates of >5% were excluded from the subsequent analyses (Supplementary Table S4). We then generated several combined datasets for different purposes of analysis, and discarded SNPs with missing rates of >5% and minor allele frequency of <5%. The summary of all datasets can be found in Supplementary Table S5.

### Statistical methods

Wright’s fixation index *F*_ST_ as a measure of population differentiation was calculated according to Weir and Hill^29^, which is an unbiased estimate that considers standardized sample sizes. Two independently integrated datasets with different numbers of SNPs were used to calculate the *F*_ST_. Population phylogenetic trees were constructed based on pairwise population *F*_ST_ metrics using a neighbor-joining method provided by PHYLIP v3.69^30^ (http://evolution.genetics.washington.edu/phylip.html), and were visualized by MEGA v6.06^31^ (http://www.megasoftware.net). The bootstrap value was based on the 1,000 repeats of *F*_ST_ calculation by randomly sampling SNPs. PCA was conducted by using EIGENSOFT v3.0^32^ (http://www.hsph.harvard.edu/alkes-price/software/). Prior to analysis, 24 SNPs were removed by LD pruning with a threshold of r^2^ = 0.8 for pairwise SNPs, and 14 individuals (4 PMM, 6 SGM, 1 IDM and 3 SLM) were filtered out with a PC1 value >2-fold standard deviation in the PCA for each independent population. Population genetic clustering was conducted by using ADMIXTURE^33^ (https://www.genetics.ucla.edu/software/admixture/) specifying K = 2 to K = 12. Cross validation (CV) was used to assess the number of clusters best-fitting the data (Supplementary Figure S4). To quantify the gene flow, we applied an LD-based method ALDER^34^ (http://groups.csail.mit.edu/cb/alder/). We used the one-reference model in ALDER, assuming only a single reference population. To circumvent the uncertainties about the exact ancestral source, we have performed the same admixture test using all the populations from each ancestral group. Because various numbers of SNPs have been identified in the populations that are used in this analysis, which ranged from ~50,000 to ~2,000,000, in the independent analysis of each donor-receptor pair, we used the SNPs shared by the pair of populations to maximize the SNP density. Similarly, when estimating the ancestral contributions of the Sri Lankan ethnic groups to Sri Lankan Malays, we examined all possible combination of the two sources (one Sri Lankan population and one Malay population). Ten individuals were randomly sampled from each population to generate a fair sample size. The supervised STRUCTURE analysis is based on the admixture model with pre-assigned ancestral individuals and two predefined clusters (K = 2). We obtained locus-specific ancestry estimations by using the linkage model implemented in STRUCTURE version 2.3^35^, with replicate runs of 20,000 burn-in iterations and 10,000 parameter-estimating iterations. The ancestral contributions to the Sri Lankan Malays were averaged across the whole genome. The PCC between the geographical coordinates and the PCA coordinates, and between the proportions of genetic components and the PCA coordinates were calculated by using R v2.11.1^36^ (http://www.r-project.org/) based on the Pearson’s correlation model.

## Additional Information

**How to cite this article**: Deng, L. *et al.* Dissecting the genetic structure and admixture of four geographical Malay populations. *Sci. Rep.* **5**, 14375; doi: 10.1038/srep14375 (2015).

## Supplementary Material

Supplementary Information

## Figures and Tables

**Table 1:** Dating gene flow to the Malays.

Group	PMM			SGM		IDM	SLM
	MY-MLY	MY-KN	MY-MN	SG-MAS	SG-MY		
European	35.93 ± 5.79	30.88 ± 6.34	31.21 ± 7.38	9.12 ± 0.68	17.96 ± 4.24	NA	7.92 ± 0.82
Southeast Asian 1	NA	261.46 ± 113.84	NA	29.53 ± 1.10	NA	NA	7.33 ± 0.59
Southeast Asian 2	NA	NA	NA	NA	NA	NA	6.41 ± 1.04
South Asian	40.21 ± 15.71	34.05 ± 3.70	35.48 ± 6.09	9.57 ± 0.89	17.48 ± 3.96	72.39 ± 18.00	8.26 ± 1.59
East Asian	6.83 ± 2.55	NA	NA	4.94 ± 0.88	NA	NA	7.47 ± 0.44

Populations in the first column are gene flow donors, and those in the first row are gene flow receptors. The date (mean ± sd) is measured for each donor-receptor pair and is measured by generations. The date of gene flow from each ancestry is the summary of the mean date estimated by the sub-populations (Supplementary Table S1). NA: No available data.
