# Supplementary material for: Dissecting the genetic structure and admixture of four geographical Malay populations
Source: Sci Rep. 2015 Sep 23;5:14375. doi: 10.1038/srep14375 (PMC4585825; doi:10.1038/srep14375)
Supplement: Supplementary Information [file srep14375-s1.doc]

**Dissecting the genetic structure and admixture of four geographical Malay populations**

*Running title:* **Genetic Diversity of Malay**

**Keywords:** Malay; Single nucleotide polymorphism; Population structure; Genetic admixture.

Lian Deng^1^, Boon-Peng Hoh^1,2^, Dongsheng Lu^1^, Woei-Yuh Saw^3,4^, Rick Twee-Hee Ong^3^, Anuradhani Kasturiratne^5^, H. Janaka de Silva^6^, Bin Alwi Zilfalil^7^, Norihiro Kato^8^, Ananda R. Wickremasinghe^5^, Yik-Ying Teo^3,4,9,10,11^, Shuhua Xu^1,12,13^,*

^1^ Chinese Academy of Sciences (CAS) Key Laboratory of Computational Biology, Max Planck Independent Research Group on Population Genomics, CAS-MPG Partner Institute for Computational Biology (PICB), Shanghai Institutes for Biological Sciences, Chinese Academy of Sciences, Shanghai 200031, China;

^2^ Faculty of Medicine and Health Sciences, UCSI University, Jalan Merana Gading, Taman Connought, 56000, Kuala Lumpur, Malaysia;

^3^ Saw Swee Hock School of Public Health, National University of Singapore, Singapore;

^4^ Life Sciences Institute, National University of Singapore, Singapore;

^5^ Department of Public Health, Faculty of Medicine, University of Kelaniya, Ragama 11010, Sri Lanka;

^6^ Department of Medicine, Faculty of Medicine, University of Kelaniya, Ragama 11010, Sri Lanka;

^7^ Department of Pediatrics, School of Medical Sciences, Universiti Sains Malaysia, Kelantan 16150, Malaysia;

^8^ Department of Gene Diagnostics and Therapeutics, National Center for Global Health and Medicine, Tokyo 1628655, Japan;

^9^ NUS Graduate School for Integrative Science and Engineering, National University of Singapore, Singapore;

^10^ Genome Institute of Singapore, Agency for Science, Technology and Research, Singapore;

^11^ Department of Statistics and Applied Probability, National University of Singapore, Singapore;

^12^ School of Life Science and Technology, ShanghaiTec University, Shanghai 200031, China;

^13^ Collaborative Innovation Center of Genetics and Development, Shanghai 200438, China.

*Correspondence and requests for materials should be addressed to S.X. ([xushua@picb.ac.cn](mailto:xushua@picb.ac.cn)).

**Supplementary Information**

**Figure and Table Legends**

**Figure S1. Neighbor-joining tree of 27 populations.**

YRI is the outgroup. The pair-wise population distance was measured by global *F*_ST_ with 1,000 bootstrapping repeats. Bootstrap values are noted on the branches. Population IDs are shown in Supplementary Table S4. Geographical groups are indicated with colors as shown in the legend. The four Malay populations are highlighted in bold font with red asterisks.

**Figure S2. Principal component analysis.**

(A) 2,546 individuals representing 81 populations from South Asia, Southeast Asia and East Asia. (B) 1,469 individuals representing 59 populations from Southeast Asia and East Asia, excluding South Asian and Southeast Asian aborigines (Malaysian Negrito and Senoi) from (A). (C) 1,451 individuals representing 58 populations from Southeast Asia and East Asia, excluding Thai Mlabri from (B). (D) 1,275 individuals representing 47 populations from Southeast Asia, excluding East Asian, Filipino Negritos, East Indonesian and Oceanian (Melanesian).

**Figure S3. Correlation between geographical coordinates and population differentiation in the Malays.**

(A) and (B) present the correlation between geographical coordinates and Malay differentiation with the vertical axis showing the value of PC1 for each individual in the Figures above, and the horizontal axis showing the populations IDs. PCA results in (A) shows 199 individuals representing 7 Malay populations, among which MY-KN and MY-MLY are combined as PMM from Kelantan, and SG-MAS and SG-MY are combined as SGM; (B) shows 156 individuals representing 5 Malay populations (excluding SLM and Minangkabau). The PC1 values are summarized in the box plot for each population. Latitudes and longitudes of the populations, as well as the Pearson’s correlation coefficient (PCC) and statistical significance (p-value) are summarized in the tables below. PMM^K^ and PMM^M^ denote Kelantan Malay and Minangkabau, respectively. Other population IDs are shown in Supplementary Table S4.

**Figure S4. Cross-validation error and population clusters of ADMIXTURE analysis.**

(A) The vertical axis represents the five-fold cross validation error of the ADMIXTURE analysis, from K = 2 to K = 12. The cross validation error is nearly stable after K > 6. In (B), each color represents an independent cluster. On all levels of K, the components of East Asian, South Asian and Southeast Asian consistently appear in the Malays. Compared to K < 9, Oceanian, Central Asian and Southeast Asian 2 decomposed from Southeast Asian 1 as the newly appeared components in K = 9, among which only Southeast Asian 2 has non-trivial contributions to the Malay populations. Southeast Asian 1 and Southeast Asian 2 are represented by aborigines from Taiwan and Malaysia, respectively, and better reflect the population genetic diversity in Southeast Asia than taking the various Southeast Asian components as a whole. On the other hand, at K > 9, the Negrito component and Southeast Asian 2 component were further decomposed, which did not provide additional insight to our understanding of the Malay populations. Therefore, we chose K = 9 as optimal for the interpretation of the admixture pattern in the Malays. Population IDs are shown in Supplementary Table S4.

**Table S1. Dating the gene flow to the Malays.**

Populations in the second column are gene flow donors, and those in the first row are gene flow receptors. The date (mean ± sd) is estimated for each donor-receptor pair and is measured by generations. NA: No available data.

**Table S2. Correlation between ancestral components and PC1 in the Malays.**

PCC (Pearson product-moment correlation coefficient) measures the correlation between the proportion of each ancestral component in the 5 Malay populations (SLM, Kelantan Malay, Minangkabau, SGM and IDM) and the values along PC1 in the PCA analysis (Supplementary Figure S2A).

**Table S3. Sri Lankan ancestral contributions (%) to the Sri Lankan Malays.**

Populations in the first column and the first row are ancestral populations, which are combined into different pairs of ancestries of the Sri Lankan Malays. In each population, we randomly sampled 10 individuals to minimize the bias caused by the inconsistent sample size.

**Table S4. Details of the populations included in the present study**

Individual QC (quality control) was conducted for each population independently. Samples with missing rate of >5% were excluded from subsequent analyses. The sample size of each population before and after QC are summarized in the third and forth columns. More detailed data information can be found in the references indicated in the fifth column.

**Table S5. Summary of datasets used in various analyses.**

^1^Population with sample size of <10 were excluded from the analysis.

^2^SNPs shared within each donor-acceptor pair were used.

^3^Ten samples were randomly selected from each population.

^4^SNPs shared within each ancestry pair were used.

**Figure S1. Neighbor-joining tree of 27 populations.**

**Figure S2. Principal component analysis.**

**Figure S3. Correlation between geographical location and population differentiation in the Malays.**

**Figure S4. Cross-validation error and population clusters of ADMIXTURE analysis.**

**Table S1. Dating the gene flow to the Malays.**

| **Group** | **Pop** | **PMM** | | |  | **SGM** | | **IDM** | **SLM** |
| --- | --- | --- | --- | --- | --- | --- | --- | --- | --- |
|  |  | **MY-MLY** | **MY-KN** | **MY-MN** |  | **SG-MAS** | **SG-MY** |  |  |
| European | CEU | 35.93 ± 5.79 | 30.88 ± 6.34 | 31.21 ± 7.38 | | 9.12 ± 0.68 | 17.96 ± 4.24 | NA | 7.92 ± 0.82 |
| Southeast Asian 1 | AX-AM | NA | 261.46 ± 113.84 | NA |  | 30.30 ± 11.55 | NA | NA | 6.91 ± 1.14 |
|  | AX-AT | NA | NA | NA |  | 28.75 ± 4.52 | NA | NA | 7.75 ± 0.90 |
| Southeast Asian2 | MY-TM | NA | NA | NA | | NA | NA | NA | 6.41 ± 1.04 |
| South Asian | GIH | 28.19 ± 6.23 | 35.77 ± 10.70 | 29.94 ± 5.01 | | 8.41 ± 0.74 | 18.94 ± 3.87 | NA | 6.84 ± 0.52 |
|  | IN-DR | 37.24 ± 9.61 | 30.85 ± 6.43 | 36.38 ± 7.14 | | 8.73 ± 1.36 | 16.49 ± 3.18 | 62.30 ± 23.13 | 6.62 ± 1.66 |
|  | IN-EL | 36.33 ± 15.52 | 34.83 ± 8.78 | 34.96 ± 6.60 | | 9.60 ± 1.80 | 16.74 ± 6.13 | 71.15 ± 33.25 | 10.25 ± 2.70 |
|  | IN-IL | 39.81 ± 13.16 | 33.95 ± 5.46 | 35.21 ± 9.77 | | 10.16 ± 2.05 | 20.50 ± 4.87 | 103.33 ± 21.71 | 7.82 ± 1.40 |
|  | IN-NI | 88.43 ± 37.57 | NA | 54.88 ± 13.18 | | 8.99 ± 4.08 | 27.88 ± 8.53 | NA | NA |
|  | IN-NL | 37.23 ± 12.84 | 34.97 ± 5.25 | 34.44 ± 8.96 | | 9.38 ± 1.87 | 20.81 ± 6.18 | NA | 6.95 ± 0.84 |
|  | IN-SP | 36.26 ± 9.86 | 37.54 ± 5.18 | 36.60 ± 8.25 | | 10.57 ± 1.62 | 14.75 ± 4.36 | NA | 9.32 ± 2.03 |
|  | IN-WI | 41.76 ± 14.73 | 42.08 ± 6.99 | 32.50 ± 7.47 | | 10.11 ± 2.19 | 17.98 ± 4.79 | 67.22 ± 24.71 | 8.83 ± 1.58 |
|  | IN-WL | 51.66 ± 12.12 | 31.04 ± 6.98 | 33.83 ± 6.57 | | 9.45 ± 1.93 | 14.48 ± 3.09 | NA | 10.79 ± 1.65 |
|  | SL-BUR | 32.16 ± 4.93 | 27.06 ± 9.61 | 32.89 ± 5.43 | | 7.81 ± 0.68 | 12.07 ± 4.79 | 57.93 ± 14.32 | NA |
|  | SL-SIN | 31.44 ± 5.05 | 33.63 ± 10.34 | 33.22 ± 6.84 | | 10.75 ± 1.19 | 15.36 ± 3.95 | NA | NA |
|  | SL-INT | 30.99 ± 4.61 | 33.94 ± 10.03 | 33.37 ± 5.46 | | 10.02 ± 0.84 | 15.76 ± 5.32 | NA | 6.89 ± 0.61 |
|  | SL-SLT | 31.17 ± 4.65 | 32.97 ± 9.51 | 33.03 ± 6.10 | | 10.43 ± 0.94 | 15.42 ± 5.16 | NA | NA |
| East Asian | CHB | 6.83 ± 2.55 | NA | NA |  | 4.28 ± 0.65 | NA | NA | 6.94 ± 0.37 |
|  | JPT | NA | NA | NA |  | 5.94 ± 1.78 | NA | NA | 7.03 ± 0.83 |
|  | JP-ML | NA | NA | NA |  | NA | NA | NA | 7.34 ± 0.91 |
|  | KR-KR | NA | NA | NA |  | 4.59 ± 1.50 | NA | NA | 7.83 ± 0.84 |
|  | CN-SH | NA | NA | NA |  | NA | NA | NA | 8.03 ± 0.86 |
|  | SG-CH | NA | NA | NA |  | NA | NA | NA | 7.63 ± 0.90 |

**Table S2. Correlation between ancestral components and PC1 in the Malays.**

| **Ancestral Population** | **South Asian** | **Central Asian** | **Southeast Asian 1** | **European** | **Southeast Asian 2** | **East Asian** | **African** | **Oceanian** | **Negrito** |
| --- | --- | --- | --- | --- | --- | --- | --- | --- | --- |
| PCC | 0.927 | 0.807 | 0.697 | 0.579 | 0.517 | 0.268 | 0.152 | 0 | 0 |
| p-value | <2.2E-16 | <2.2E-16 | <2.2E-16 | <2.2E-16 | 1.09E-14 | 1.03E-4 | 0.020 | 0.769 | 0.803 |

**Table S3. Sri Lankan ancestral contributions (%) to the Sri Lankan Malays.**

| **Ancestral Population** | **PMM** | | |  | **SGM** | | **IDM** |
| --- | --- | --- | --- | --- | --- | --- | --- |
|  | **MY-MLY** | **MY-KN** | **MY-MN** |  | **SG-MAS** | **SG-MY** |  |
| SL-BUR | 60.6 | 56.9 | 58.5 | | 57.7 | 57.9 | 60.9 |
| SL-SIN | 58.1 | 55.7 | 57.6 | | 56.9 | 55.1 | 59.7 |
| SL-SLT | 57.6 | 55.4 | 57.2 | | 55.4 | 55.7 | 59.3 |
| SL-INT | 57.9 | 55.5 | 57.4 | | 54.9 | 55.9 | 60.3 |

**Table S4. Details of the populations included in the present** study.

| **Population ID** | **Details** | **No. Samples before QC** | **No. Samples after QC** | **Reference** |
| --- | --- | --- | --- | --- |
| YRI | Yoruba in Ibadan, Nigeria | 116 | 116 | [11] |
| CEU | Utah residents with Northern and Western European ancestry from the CEPH collection | 112 | 112 | [11] |
| GIH | Gujarati Indians in Houston, Texas | 88 | 88 | [11] |
| CHD | Chinese in Metropolitan Denver, Colorado | 85 | 85 | [11] |
| CHB | Han Chinese in Beijing, China | 84 | 84 | [11] |
| JPT | Japanese in Tokyo, Japan | 86 | 86 | [11] |
| PK-HZ | Hazara in Pakistan | 22 | 22 | [12] |
| PK-BR | Brahui in Pakistan | 25 | 25 | [12] |
| PK-BA | Balochi in Pakistan | 24 | 24 | [12] |
| PK-SI | Sindhi in Pakistan | 24 | 24 | [12] |
| PK-HU | Hunza Burusho in Pakistan | 25 | 25 | [12] |
| PK-KL | Kalash in Pakistan | 23 | 23 | [12] |
| PK-MK | Makrani in Pakistan | 25 | 25 | [12] |
| PK-PT | Pathan in Pakistan | 22 | 22 | [12] |
| Cambodian | Cambodian in Cambodia | 10 | 10 | [12] |
| Papuan | Papuan in New Guinea | 17 | 17 | [12] |
| SG-CHS | Chinese in Singapore | 96 | 96 | [14] |
| SG-MAS | Malay in Singapore | 89 | 89 | [14] |
| SG-INS | Indian in Singapore | 83 | 83 | [14] |
| MY-MLY | Malay in Kelantan, Malaysia | 17 | 17 | [5] |
| MY-PML | Proto-Malay in Malaysia | 4 | 4 | [5] |
| MY-SNI | Senoi in Malaysia | 17 | 17 | [5] |
| MY-NGO | Negrito in Malaysia | 46 | 22 | [5] |
| SL-MLY | Malay in Sri Lanka | 27 | 27 | - |
| SL-BUR | Burger in Sri Lanka | 35 | 35 | - |
| SL-SIN | Sinhalese in Sri Lanka | 200 | 200 | - |
| SL-INT | Tamil in Sri Lanka with Indian ancestry | 200 | 200 | - |
| SL-SLT | Tamil in Sri Lanka | 103 | 103 | - |
| AX-AM | Ami in Taiwan, China | 10 | 10 | [8] |
| AX-AT | Atayal in Taiwan, China | 10 | 10 | [8] |
| AX-ME | Melanesian in Pacific | 5 | 5 | [8] |
| CN-CC | Zhuang in China | 26 | 26 | [8] |
| CN-GA | Han Chinese in Guangdong, China | 30 | 30 | [8] |
| CN-HM | Hmong in China | 26 | 26 | [8] |
| *(Continued)* | | | | |

| **Population ID** | **Details** | **No. Samples before QC** | **No. Samples after QC** | **Reference** |
| --- | --- | --- | --- | --- |
| CN-JN | Jinuo in China | 29 | 29 | [8] |
| CN-SH | Han Chinese in Shanghai, China | 21 | 19 | [8] |
| CN-UG | Uyghur in China | 26 | 26 | [8] |
| CN-WA | Wa in China | 55 | 55 | [8] |
| ID-AL | Alorese in Indonesia | 19 | 19 | [8] |
| ID-DY | Dayak in Indinesia | 12 | 12 | [8] |
| ID-JA | Javanese in Indonesia | 34 | 34 | [8] |
| ID-JV | Javanese in Indonesia | 18 | 18 | [8] |
| ID-KR | Batak Karo in Indonesia | 17 | 17 | [8] |
| ID-LA | Lamaholot in Indonesia | 20 | 20 | [8] |
| ID-LE | Lembata in Indonesia | 19 | 19 | [8] |
| ID-ML | Malay in Indonesia | 12 | 11 | [8] |
| ID-MT | Mentawai in Indonesia | 15 | 15 | [8] |
| ID-RA | Manggarai in Indonesia | 17 | 17 | [8] |
| ID-SB | Kambera in Indonesia | 20 | 20 | [8] |
| ID-SO | Manggarai in Indonesia | 19 | 19 | [8] |
| ID-SU | Sundanese in Indonesia | 25 | 25 | [8] |
| ID-TB | Batak in Indonesia | 20 | 20 | [8] |
| ID-TR | Toraja in Indonesia | 19 | 19 | [8] |
| IN-DR | Upper-caste in India | 24 | 24 | [8] |
| IN-EL | Upper-caste in India | 16 | 15 | [8] |
| IN-IL | Upper-caste in India | 15 | 14 | [8] |
| IN-NI | Tharu in India | 20 | 20 | [8] |
| IN-NL | Upper-caste in India | 15 | 11 | [8] |
| IN-SP | Upper-caste in India | 22 | 22 | [8] |
| IN-TB | Ladakhi in India | 23 | 23 | [8] |
| IN-WI | Bhil in India | 24 | 24 | [8] |
| IN-WL | Upper-caste in India | 14 | 14 | [8] |
| JP-ML | Japanese in Japan | 71 | 71 | [8] |
| JP-RK | Ryukyuan in Japan | 49 | 49 | [8] |
| KR-KR | Korean in Korea | 90 | 83 | [8] |
| MY-BD | Bidayuh in Malaysia | 49 | 47 | [8] |
| MY-JH | Negrito (Jehai) in Malaysia | 50 | 50 | [8] |
| MY-KN | Malay in Kelantan, Malaysia | 18 | 13 | [8] |
| MY-KS | Negrito (Kensiu) in Malaysia | 30 | 30 | [8] |
| MY-MN | Malay (Minangkabau) in Malaysia | 20 | 19 | [8] |
| MY-TM | Proto-Malay (Temuan) in Malaysia | 49 | 44 | [8] |
| *(Continued)* | | | | |

| **Population ID** | **Details** | **No. Samples before QC** | **No. Samples after QC** | **Reference** |
| --- | --- | --- | --- | --- |
| PI-AE | Negrito in the Philippines | 8 | 2 | [8] |
| PI-AG | Negrito in the Philippines | 8 | 3 | [8] |
| PI-AT | Negrito in the Philippines | 23 | 9 | [8] |
| PI-IR | Negrito in the Philippines | 9 | 2 | [8] |
| PI-MA | Manobo in the Philippines | 18 | 11 | [8] |
| PI-MW | Negrito in the Philippines | 19 | 3 | [8] |
| PI-UB | Urban in the Philippines | 20 | 20 | [8] |
| PI-UI | Urban in the Philippines | 20 | 20 | [8] |
| PI-UN | Urban in the Philippines | 18 | 18 | [8] |
| SG-CH | Han Chinese in Singapore | 29 | 29 | [8] |
| SG-ID | Tamil in Singapore with Indian ancestry | 30 | 30 | [8] |
| SG-MY | Malay in Singapore | 30 | 30 | [8] |
| TH-HM | Hmong in Thailand | 19 | 19 | [8] |
| TH-KA | Karen in Thailand | 20 | 19 | [8] |
| TH-LW | Lawa in Thailand | 19 | 19 | [8] |
| TH-MA | Mlabri in Thailand | 18 | 18 | [8] |
| TH-MO | Mon in Thailand | 19 | 18 | [8] |
| TH-PL | Palong in Thailand | 18 | 18 | [8] |
| TH-PP | Plang in Thailand | 18 | 18 | [8] |
| TH-TK | Tai Kern in Thailand | 18 | 18 | [8] |
| TH-TL | Tai Lue in Thailand | 20 | 19 | [8] |
| TH-TN | H'Tin in Thailand | 17 | 16 | [8] |
| TH-TU | Tai Yuan in Tailand | 20 | 20 | [8] |
| TH-TY | Tai Yong in Tailand | 18 | 18 | [8] |
| TH-YA | Yao in Thailand | 19 | 18 | [8] |
| TW-HA | Han Chinese in Taiwan, China | 48 | 48 | [8] |
| TW-HB | Han Chinese in Taiwan, China | 32 | 32 | [8] |

| **Dataset** | **No. Samples** | **No. SNPs** | **Analysis** |
| --- | --- | --- | --- |
| HapMap + SGVP + PASNP + Sri Lankan dataset + Malaysian ethnicities dataset | 3025^1^ | 7012 | Population phylogeny (Figure 1) |
| HapMap + SGVP + HGDP + Sri Lankan dataset + Malaysian ethnicities dataset | 1677^1^ | 90634 | Population phylogeny (Supplementary Figure S1) |
| HapMap + SGVP + PASNP + Sri Lankan dataset + Malaysian ethnicities dataset | 3053 | 3240 | PCA (Figure 2, Supplementary Figure S2, Supplementary Figure S3) |
| HapMap + SGVP + HGDP + PASNP + Sri Lankan dataset + Malaysian ethnicities dataset | 2604 | 4894 | ADMIXTURE clustering analysis (Figure 3, Supplementary Figure S4) |
| HapMap + SGVP + PASNP + Sri Lankan dataset + Malaysian ethnicities dataset | 1414 | 14720-1159620^2^ | Gene flow estimation (Table 1, Supplementary Table S1) |
| SGVP + PASNP + Sri Lankan dataset + Malaysian ethnicities dataset | 100^3^ | 5209-8299^4^ | Supervised STRUCTURE analysis (Supplementary Table S3) |

**Table S5. Summary of datasets used in various analyses.**
